# BEING A SUPERWOMAN, THE EXPLORATION OF AFRICAN AMERICAN WOMEN DEMENTIA CAREGIVERS AND THEIR MENTAL HEALTH

**DOI:** 10.1093/geroni/igae098.4342

**Published:** 2024-12-31

**Authors:** Shanae Rhodes

**Affiliations:** University of Texas Health Science Center San Antonio, San Antonio, Texas, United States

## Abstract

Existing literature presents evidence that African American caregivers experience a health paradox where they have commonly reported having worse physical health but better mental health related to caregiver burden when compared to other racial groups. However, there are inconsistencies across the research literature regarding mental health outcomes for this population. Guided by Pearlin’s Stress Process Model and the Superwoman Schema, the purpose of this study was to explore the influence of the Superwoman Schema characteristics and caregiver burden on the psychological health (anxiety and depressive symptoms) of African American female caregivers. A mixed methods design was used for this study. This presentation will focus on the quantitative segment. Cross-sectional correlations and regression analyses were conducted using data collected from 127 participants to investigate the relationship between caregiver burden, the five Superwoman characteristics, and psychological health for African American female caregivers. Results showed that caregivers who reported a higher endorsement of any of the Superwoman characteristics were more likely to report higher caregiver burden, anxiety, and depressive symptoms. Endorsement of some of the Superwoman characteristics (i.e., obligation to present an image of strength and obligation to help) partially mediated the relationship between caregiver burden and anxiety and caregiver burden and depressive symptoms. Future research should continue to examine the mediation of Superwoman characteristics on the relationship between caregiver burden and psychological health outcomes. These partial mediators can inform intervention development to mitigate the negative impact of caregiver burden on mental health.

